# Microbial diversity in biodeteriorated Greek historical documents dating back to the 19th and 20th century: A case study

**DOI:** 10.1002/mbo3.596

**Published:** 2018-02-27

**Authors:** Kiriaki Karakasidou, Katerina Nikolouli, Grigoris D. Amoutzias, Anastasia Pournou, Christos Manassis, George Tsiamis, Dimitris Mossialos

**Affiliations:** ^1^ Department of Biochemistry & Biotechnology University of Thessaly Larissa Greece; ^2^ Department of Conservation of Antiquities and Works of Art Technological Educational Institute of Athens Athens Greece; ^3^ Department of Environmental and Natural Resources Management University of Patras Agrinio Greece

**Keywords:** 16S rRNA, historical documents, ITS, microbial diversity, paper degradation, phylogeny

## Abstract

Paper documents in archives, libraries, and museums often undergo biodeterioration by microorganisms. Fungi and less often bacteria have been described to advance paper staining, so called “foxing” and degradation of paper substrates. In this study, for the first time, the fungal and bacterial diversity in biodeteriorated paper documents of Hellenic General State Archives dating back to the 19th and 20th century has been assessed by culture‐dependent and independent methods. The internally transcribed spacer (ITS) region and 16S rRNA gene were amplified by PCR from fungal and bacterial isolates and amplicons were sequenced. Sequence analysis and phylogeny revealed fungal phylotypes like *Penicillium* sp*., Cladosporium* sp*., Penicillium citrinum, Alternaria infectoria, Alternaria alternata, Epicoccum nigrum,* and *Penicillium chrysogenum* which are often implicated in paper deterioration. Bacterial phylotypes closely related to known biodeteriogenic bacteria such as *Bacillus* spp., *Micrococcus* spp., *Kocuria* sp. in accordance with previous studies were characterized. Among the fungal phylotypes described in this study are included well‐known allergens such as *Penicillium* spp., *Alternaria* spp., and *Cladosporium* spp. that impose a serious health threat on staff members and scholars. Furthermore, fungal isolates such as *Chalastospora gossypii* and *Trametes ochracea* have been identified and implicated in biodeterioration of historical paper manuscripts in this study for the first time. Certain new or less known fungi and bacteria implicated in paper degradation were retrieved, indicating that particular ambient conditions, substrate chemistry, or even location might influence the composition of colonizing microbiota.

## 1 INTRODUCTION

Historical documents and archives are cultural heritage objects of great importance and their proper preservation is a major concern. They provide a kind of collective memory used by scholars all over the world to study different historic periods. These documents are composed of organic substrates such as paper, parchment, papyrus, or photographic paper and they are commonly preserved in libraries, archives, and museums. Such items can be considered as substrates hosting a reservoir of microorganisms, the majority of which is involved in their biodeterioration. Therefore, it is crucial to preserve them in conditions which inhibit microbiota proliferation and to develop suitable monitoring schemes to avoid their damage (Kraková et al., 2016; Sterflinger & Pinzari, 2012). Paper materials in indoor environments suffer from various physicochemical and biological agents, while most of them are subject to biodeterioration caused by fungi and bacteria (Pasquarella et al., 2012). Biodeterioration of paper results in undesirable and irreversible changes in the physicochemical and mechanical properties of historical documents (Lavin, de Saravia, & Guiamet, 2014). Fungi are considered major agents of biodeterioration and more than 200 fungal species have been isolated from paper documents, books, and prints (Pinzari, Pasquariello, & De Mico, 2006). Fungi can damage historical paper documents by either producing cellulolytic enzymes or by releasing weak acids and pigments (Arai, 2000; El Bergadi, Laachari, Elabed, Mohammed, & Ibnsouda, 2014; Pinzari, Cialei, & Barbabietola, 2010; Sterflinger, 2010; Zotti, Ferroni, & Calvinic, 2011). Cellulolytic enzymes can degrade cellulose microfibrils and under favorable conditions paper material is decayed in short time (El Bergadi et al., 2014; Sterflinger, 2010). Excretion of weak acids and pigments creates “rusty” stains and discolorations on the outer paper surface, a phenomenon known as *foxing* (Arai, 2000; El Bergadi et al., 2014; Zotti et al., 2011). Following fungal colonization and biodegradation, degraded cellulose microfibrils offer an enriched substrate for bacterial growth (Michaelsen, Piñar, & Pinzari, 2010). Bacterial microflora has been reported to colonize and damage paper material (Cappitelli, Pasquariello, Tarsitani, & Sorlini, 2010; Kraková et al., 2012; Lavin et al., 2014). For instance, *Bacillus* spp. which have cellulolytic and proteolytic activity are implicated in biodeterioration and are commonly isolated from foxed paper documents (De Paolis & Lippi, 2008; Kraková et al., 2012; Lavin, de Saravia, & Guiamet, 2016; Lavin et al., 2014). Nevertheless, *a Bacillus licheniformis* strain has been recently isolated from 19th‐century paper documents that secretes a 20 kDa protein active against common biodeteriogenic fungi (Jacob, Bhagwat, & Kelkar‐Mane, 2015). Another major issue involved in the deterioration of library material by microorganisms is the health impact on librarians and users. Several fungal species that might produce mycotoxins with an effect on the skin and/or the respiratory system have been isolated from paper documents and archives (Mesquita et al., 2009; Sterflinger & Pinzari, 2012). Considering the importance of cultural heritage and the potential health impact on library workers, conservators, and visitors, the development of efficient monitoring and management tools is deemed necessary.

Poor storage conditions advance the deterioration state of various paper manuscripts. Microbial attack affects the whole paper object, starting from the surface and progressively penetrating through all layers. Several physicochemical methods have been employed to treat contaminated objects in archives and museums and conserve them in order to prevent further deterioration. Most commonly used strategies include, gamma rays, the use of biocides such as calcium propionate, essential oils, parabens, titanium oxide nanoparticles, and fumigation with ethylene oxide (Michaelsen, Pinzari, Barbabietola, & Piñar, 2013; Sequeira, Cabrita, & Macedo, 2012; Sequeira, Phillips, Cabrita, & Macedo, 2017). Temperature and humidity conditions have been proven as crucial factors that can enhance the degrading activity of microorganisms (Montemarini‐Corte, Ferroni, & Salvo, 2003) as their proper control can prevent the development of cellulolytic fungi and bacteria (Sterflinger, 2010). Although climate control and frequent cleaning have been used as front‐line methods to control fungal and bacterial contamination leading to biodeterioration, there is a growing interest for target‐specific approaches. Therefore, knowing the type of organisms colonizing paper material and their metabolic activities are critical factors that can assist curators and conservators to select the most efficient and feasible disinfection method. The most frequently used methods of studying microbial biodeterioration involve invasive sampling and culture approaches. However, culture‐dependent methods might cause additional surface damage of the document and suffer from low sensitivity since many candidate species are noncultivable (actually less than 1%) (Cappitelli et al., 2010; Mueller & Schmit, 2007; Piñar, Tafer, Sterflinger, & Pinzari, 2015). Furthermore, they are also strongly affected by the airborne fungal spores which are ubiquitous in the air and might be easily attached on the paper thus detecting and identifying fungal species that are not actually implicated in the foxing process (Choi, 2007).

On the other hand, metagenomics (culture‐independent methods) are an alternative approach to investigate the involvement of microorganisms in the biodeterioration process. Metagenomics are widely adopted to identify unculturable or yet‐uncultured microbes which are part of microbial communities present virtually in any environment (Nikolouli & Mossialos, 2012), including paper documents, thus allowing a better insight in the biodeterioration process.

The aim of this work was to study the fungal and bacterial diversity in biodeteriorated paper manuscripts dating back to the 19th and 20th century, stored in the Hellenic General State Archives (Athens). These manuscripts are of significant historic importance since they are dating from the Greek postrevolutionary period and include information regarding the constitution of the Independent Greek State and following historic periods. Unfortunately, these documents are severely decayed and a target‐specific preventive conservation strategy is necessary, able to monitor them for avoiding further biodeterioration. Identification of the microbial diversity present in these manuscripts, possibly implicated in biodeterioration, has been performed by culture‐dependent and independent techniques.

## 2 MATERIALS AND METHODS

### 2.1 Sampling and microbial culture conditions

**Figure 1:**
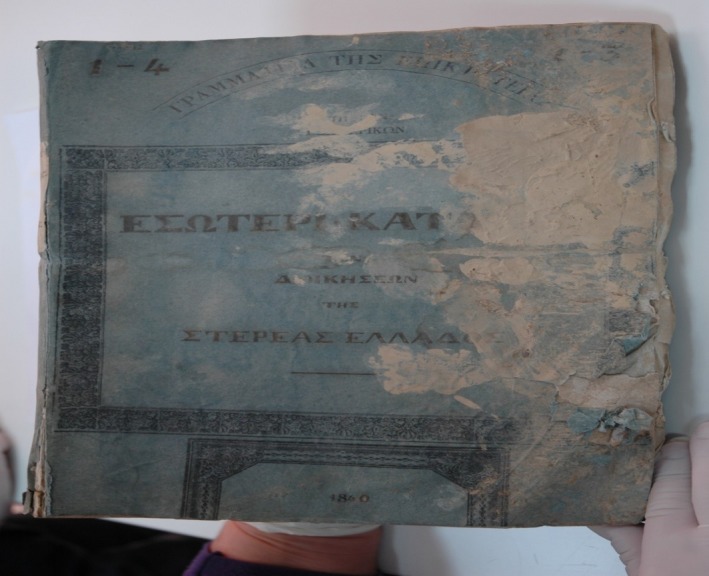
This historical document dating back to 1840 has shown extensive signs of biodeterioration upon macroscopic examination (Sample 4). Image courtesy of Hellenic General State Archives (Athens, Greece)

Five historical paper documents were offered for analysis by the Hellenic General State Archives (Athens, Greece). Four out of five documents dated back to 19th century (1840–1843), while the fifth one dated back to 20th century (1919). The samples were macroscopically examined and selected for further analysis, based on macroscopic patterns of biodeterioration such as discoloration, permanent staining, structural damage, and musty odor (Figure 1). Two sampling strategies were employed. In the first one, samples were collected from documents (surface area circa 30 cm^2^) demonstrating macroscopic biodeterioration patterns using sterile cotton swabs, whereas in the second one, sterile scalpels were used to remove small fragments (circa 0.5 cm^2^) directly from a heavily biodegraded area of documents. Both cotton swabs and paper fragments were kept at 4°C, in sterile vials till use. Cotton swabs were then used to inoculate Malt Yeast Extract agar plates (Lab M, UK) containing streptomycin (500 μg/ml) (Serva, Germany) for fungal or Nutrient Agar plates (Lab M, UK) for bacterial isolation. Agar plates were incubated at 30°C up to 7 days or up to 3 days, respectively. All fungal and bacterial isolates were kept at −80°C as glycerol stocks.

### 2.2 DNA extraction and PCR amplification from microbial isolates

DNA was extracted from 17 morphologically distinct fungal isolates using the NucleoSpin Plant II Kit (Macherey‐Nagel, GERMANY) according to manufacturer's instructions. Bacterial DNA was extracted from 15 bacterial isolates as described before (Spilker, Coenye, Vandamme, & LiPuma, 2004). Briefly, a single CFU was suspended in 20 μl of lysis buffer containing 0.25% (v/v) sodium dodecyl sulfate (Serva, GERMANY) and 0.05 N NaOH (Serva, GERMANY). After heating for 15 min at 95°C, 180 μl sterile H_2_O was added and lysis suspension was stored at −20°C up to 1 week.

The following primer sets were used for the PCR amplification of the internally transcribed spacer (ITS) region in fungi: ITS1‐ITS4 (for 15 isolates) and ITS1F‐NLB4 (for 2 isolates) (Table S1).

The amplification reaction mixture contained: 1U ExTaq DNA polymerase (Takara, JAPAN), 1× PCR buffer, 0.4 μmol/L of each primer, 250 μmol/L dNTPs, 1 μl DNA template, and deionized sterile water to a final volume of 50 μl.

PCR conditions were based on the protocol described by Mesquita et al. (2009): initial denaturation at 95°C for 2 min, followed by 40 cycles of denaturation at 95°C for 1 min, annealing at 55°C for 1 min, and extension at 72°C for 1 min. A final extension step of 72°C for 10 min was added. PCR products were purified from primers, nucleotides, and salts using the Nucleospin Extract II kit (Macherey‐Nagel, GERMANY).

The universal bacterial primers 27F and 1492R were used for the amplification of the bacterial 16S rRNA gene (Table S1). The amplification reaction mixture contained: 1U ExTaq DNA polymerase (Takara, JAPAN), 1× PCR buffer, 0.4 μmol/L of each primer, 250 μmol/L dNTPs, 6 μl DNA template, and deionized sterile water to a final volume of 25 μl. PCR conditions were: initial denaturing step at 94°C for 5 min, followed by 30 cycles of denaturation at 94°C for 1 min, annealing at 57°C for 30 s, and extension at 72°C for 90 s. A final extension step of 72°C for 10 min was added. PCR products were purified from primers, nucleotides, and salts using the Nucleospin Extract II kit (Macherey‐Nagel, GERMANY) according to manufacturer's instructions.

### 2.3 DNA extraction directly from paper samples and PCR amplification

Total DNA extraction directly from all paper samples was performed using the Nucleospin Soil Kit (Macherey‐Nagel, GERMANY) according to manufacturer's instructions but it was successful only for samples 1 and 4 as it was assessed by 0.8% agarose gel. The primers ITS1F and NLB4 were used for the PCR amplification of the ITS region in both DNA samples. DNA from sample 4 was also used to amplify the bacterial 16S rRNA gene with the universal primers 27F and 1492R.

PCR reactions both for fungi and bacteria were performed according to the protocols described in section 2.2. PCR products were purified from primers, nucleotides, and salts using the Nucleospin Extract II kit (Macherey‐Nagel, GERMANY) according to manufacturer's instructions.

### 2.4 Construction of ITS and 16S rRNA gene clone libraries and sequence analysis

ITS amplicons from samples 1 and 4 were used for the construction of two distinct clone libraries, while the bacterial clone library was constructed using the 16S rRNA amplicons from sample 4. PCR amplicons were cloned in the pGEM‐T Easy vector (Promega, USA) following the manufacturer's instructions. Clone libraries were kept at −80°C as glycerol stocks. Plasmid DNA was prepared for sequencing from 44 clones of the first ITS library (sample 1), 37 clones of the second ITS library (sample 4), and 64 clones of the bacterial 16S rRNA gene library using the Nucleospin Plasmid kit (Macherey‐Nagel, GERMANY) following the manufacturer's instructions. Sequencing was performed using an ABI310 sequencer (ANTISEL Selidis Bros SA, Greece). Good's C estimator [1 − (*n*_1_/*N*)] was used to calculate ITS library coverage (Good, 1953), where n_1_ is the number of OTUs (operational taxonomic unit) represented by only one clone and N is the total number of clones examined in each library (Chao, 1984).

The index of diversity was estimated by *S*_Chao1_:

$$S_{\mathrm{Chao}1}=S_{\mathrm{obs}}+n_{1}\left(n_{1}-1\right)/2\left(n_{2}+1\right),\;\;\text{where}$$

*S*_obs_ is the number of OTUs observed in the library, while *n*_1_ and *n*_2_ are the number of OTUs occurring one and two times, respectively (Chao, 1987). The confidence interval (CI) for *S*_Chao1_ index estimation is 95%.

Bacterial sequences were checked for chimeras using usearch, (Edgar, Haas, Clemente, Quince, & Knight, 2011) and chimeras were excluded from further analysis. All derived sequences were aligned using the program MEGA64 v.5 (Tamura, Dudley, Nei, & Kumar, 2007). The obtained ITS and 16S rRNA gene sequences were submitted in BLAST search of the NCBI database (http://blast.ncbi.nlm.nih.gov/). Furthermore, 16S rRNA gene sequences were submitted in the RDP database (http://rdp.cme.msu.edu/). OTU (Operational Taxonomic Unit), Chao1 species estimator, and Shannon index for the 16S rRNA gene library were calculated using Mothur with the average neighbor assignment algorithm (Schloss et al., 2009). The DNA sequences reported in this study have been deposited in GenBank with accession numbers KC492563‐KC492579 for fungal isolates, KC492510‐KC492519 and KC4492521‐KC492525 for bacterial isolates, KC920851‐KC920890 for ITS clone libraries, and KC492526‐KC492562 for 16S rRNA clone library.

### 2.5 Construction of phylogenetic trees

Reference bacterial and fungal sequences for the 16S rRNA and ITS region were retrieved with MOLE‐BLAST (https://blast.ncbi.nlm.nih.gov/moleblast/moleblast.cgi), using as queries the sequences that were sequenced by this project. MOLE‐BLAST is a specialized tool specifically designed for classifying prokaryotic 16S rRNA and fungal ITS sequences. For each query sequence, the best reference sequence from the MOLE‐BLAST database was retrieved. The query and reference sequences were aligned with Muscle (Edgar, 2004), within the Seaview software (Gouy, Guindon, & Gascuel, 2010). Fungal ITS sequences were aligned separately, whereas some of the bacterial 16S rRNA sequence fragments had very little or no overlap with the other bacterial sequences. Thus, two separate bacterial multiple alignments and phylogenetic trees were generated.

For each of the three alignments, the Generalized Time Reversible (GTR) substitution model was selected by JModelTest2 (Darriba, Taboada, Doallo, & Posada, 2012). Next, Maximum Likelihood phylogenetic trees were generated within the Seaview software (model: GTR; 4 categories of rate variation; nucleotide equilibrium frequencies: empirical; invariable sites: optimized; tree searching operations: SPR; starting tree: BioNJ). Each of the three phylogenetic trees were visualized with the Figtree software (http://tree.bio.ed.ac.uk/software/figtree/).

## 3 RESULTS AND DISCUSSION

In this study, the microbial diversity of biodeteriorated Greek historical documents was assessed by culture‐dependent and independent methods for the first time. In total, 17 distinct fungal phylotypes, belonging to Ascomycetes and Basidiomycetes were identified by culture‐dependent methods. These were closely related to: *Penicillium* sp. (2), *P. citrinum* (2)*, Epicoccum nigrum* (1), *Cladosporium* sp. (2), *P. chrysogenum (*2), *Chalastospora gossypii* (*Alternaria malosum*) (1), *Talaromyces flavus* (1), *Trametes ochracea* (1), *Lewia infectoria (Alternaria infectoria*) (1) as well as four uncultured fungal clones (Table 1). Two ITS clone libraries were also constructed to assess fungal diversity by culture‐independent methods. Sequence analysis of the sample 1 clone library has revealed that the predominant fungal phylotype was closely related to ascomycete *Alternaria* sp. (43 out of 44 clones), while a second phylotype was closely related to *P. chrysogenum* (1 out of 44 clones). Library coverage analysis based on Good's C estimator was estimated at 97.7%, while the diversity index S_Chao1_ confirmed the low fungal diversity present in this clone library (Table 3). Similarly, sequence analysis of the second ITS clone library has revealed that the predominant fungal phylotype was closely related to *A. alternata* (35 out of 37 clones)*,* while phylotypes closely related to *P. chrysogenum* and *Candida* sp. were identified less frequently (1 clone each). Library coverage analysis based on Good's C estimator was estimated at 94.6%, while the diversity index *S*_Chao1_ confirmed the low fungal diversity present in this clone library (Table 3).

**Table 1:** Fungi isolated and identified with culture‐dependent methods

Strain/accession number	Paper document	Closest homologue accession number & sequence similarity (% identity)
FC1/KC492563	Sample 2	FJ647577.1 *Penicillium* sp. (99.8%)
FC2/KC492564	Sample 1	FJ820627.1 Uncultured fungus clone (96%)
FC3/KC492565	Sample 2	KU375629.1 *Lewia infectoria (Alternaria infectoria)* (100%)
FC4/KC492566	Sample 2	JN206678.1 *Penicillium citrinum* (99.8%)
FC5/KC492567	Sample 4	EF505595.1 Uncultured fungus clone (98%)
FC6/KC492568	Sample 4	GQ999287.1 Uncultured fungus clone (100%)
FC7/KC492569	Sample 5	MF925489.1 *Epicoccum nigrum* (100%)
FC8/KC492570	Sample 5	JN689952.1 *Cladosporium* sp. (100%)
FC9/KC492571	Sample 1	GU054202.1 Uncultured fungus clone (100%)
FC10/KC492572	Sample 4	JN032681.1 *Penicillium chrysogenum* (100%)
FC11/KC492573	Sample 3	JN226938.1 *Penicillium* sp. (100%)
FC12/KC492574	Sample 2	JN986785.1 *Penicillium chrysogenum* (99.8%)
FC13/KC492575	Sample 3	HQ696055.1 *Cladosporium* sp. (100%)
FC14/KC492576	Sample 1	KR150257.1 *Penicillium citrinum* (100%)
FC15/KC492577	Sample 4	GU183130 *Chalastospora gossypii* (*Alternaria malosum*) (96.2%)
FC16/KC492578	Sample 4	EF123253.1 *Talaromyces flavus* (99.8%)
FC17/KC492579	Sample 4	AB158314.1 *Trametes ochracea* (99.7%)

**Figure 2:**
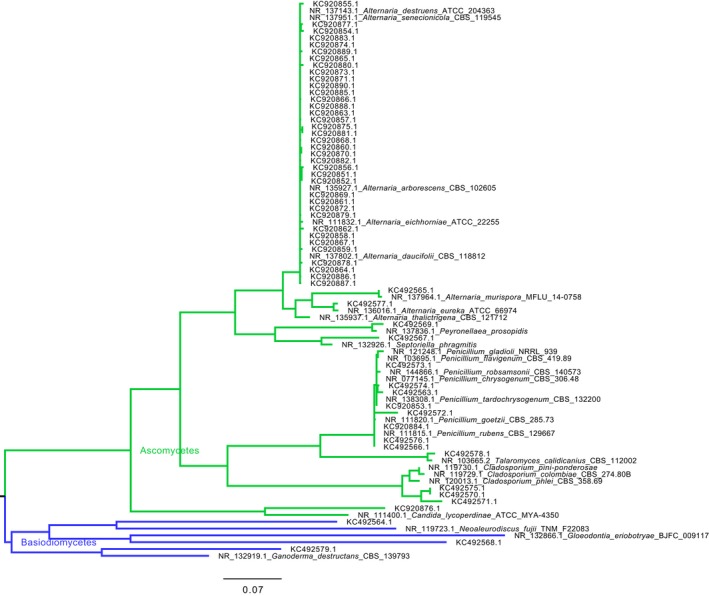
Maximum Likelihood Phylogenetic tree of the fungal ITS sequences identified in this study, together with closely related sequences from publicly available sequence databases, retrieved by MOLE‐BLAST

The phylogenetic tree presented in Fig 2 depicts the phylogenetic proximity between the fungal phylotypes retrieved by culture‐dependent and independent methods in this study and their most closely related fungi in the MOLE‐BLAST public database. Thirty‐nine sequences were clustered within the *Alternaria* genus. One sequence classified by BLAST analysis as *Epicoccum nigrum* was closely related to *Peyronellaea prosopidis,* within *Didymellaceae* group. One sequence which was rather divergent from *Septoriella phragmitis*, most probably belongs to *Phaeosphaeriaceae* group based on subsequent BLAST analysis. Eight sequences were within the *Penicillium* genus. One sequence classified as *Talaromyces flavus* by BLAST analysis was very closely related to *T. calidicanius*. Three sequences were clustering together and were a close sister group to *Cladosporium*. One sequence which was identified by BLAST analysis as *Candida* sp. was moderately distant to *C. lycoperdinae*. Thus, for the Ascomycetes, the phylogenetic tree was in agreement with the initial BLAST analysis alone. In the Basidiomycetes group, one sequence was a rather distant relative of *Neoaleurodiscus fujii*. Subsequent BLAST analysis revealed that this particular sequence was 96% identical to *Stereum rugosum*. Another sequence was a distant relative of *Gloeodontia eriobotryae*. Subsequent BLAST analysis revealed that this particular sequence was 100% identical to *Peniophora* sp. Finally another sequence was a rather distant relative to *Ganoderma destructans*. Nevertheless, BLAST analysis revealed that this particular sequence was 99.7% identical to *Trametes ochracea*. Most probably, the poor representation of this type of sequences from Basiodiomycetes in the MOLE‐BLAST database is responsible for not identifying very close relatives in this particular evolutionary lineage.

Typical fungal colonizers of paper documents are found to be species of slow‐growing ascomycetes as well as mitosporic xerophilic fungi like *Aspergillus* spp., *Penicillium* spp, and *Cladosporium* spp. (Pinzari et al., 2006; Polo, Cappitelli, Villa, & Pinzari, 2017). Fungi isolated in this study are often implicated in biodeterioration of historical documents (Kraková et al., 2012, 2016; Lech, 2016; Mesquita et al., 2009; Paiva de Carvalho et al., 2016; Polo et al., 2017; Sterflinger & Pinzari, 2012). Interestingly, isolated fungi included phylotypes closely related to *Trametes ochracea* and *Chalastospora gossypii,* which to the best of our knowledge were never identified in biodeteriorated paper documents before this study. *T. ochracea* is a well‐known wood‐decaying basidiomycete (Dai, Xu, Yang, & Jiang, 2008; Olennikov, Agafonova, Penzina, & Borovskiî, 2014) which excretes enzymes implicated in lignin degradation. Therefore, its implication in paper document biodeterioration is very likely. *Talaromyces flavus* is a slow‐growing endophytic fungus with the potential to be used as biocontrol agent and in bioremediation of the commonly used herbicide, nicosulfuron (Song et al., 2013; Yuan et al., 2017). *T. flavus* has been recently isolated from a photo conserved in the National Archives of Cuba (Borrego & Perdomo, 2014). The closely related species *T. rugulosus* has been recently isolated from wooden organ pipes and it has demonstrated cellulolytic and lignolytic activity (Štafura et al., 2017). Interestingly, another related species *T*. *helicus*, has been isolated from a map and it has demonstrated amylolytic and proteolytic activities that could justify it as a potential cellulose degrader (Borrego, Lavin, Perdomo, Gómez de Saravia, & Guiamet, 2012; Guiamet, Borrego, Lavin, Perdomo, & Gómez de Saravia, 2011). *C. gossypii* (synonym of *A. malorum*) is a rather obscure ascomycete, which is poorly studied (Crous et al., 2009). Its implication in biodeterioration of cultural objects is not known at all.

Moreover, a fungal phylotype closely related to *Candida* sp. was identified in this study. Interestingly, in a study using a similar approach, 17% of ITS clones were identified within the genus *Candida,* but no evidence regarding implication in paper deterioration was provided (Principi, Villa, Sorlini, & Cappitelli, 2011). Recently, a fungal phylotype closely related to *Candida* sp. was isolated from photos conserved in the National Archives of Cuba but it was neither able to degrade paper nor crystalline cellulose (Borrego, Molina, & Santana, 2015).

Among the fungal phylotypes retrieved in this study, well‐known allergens such as *Penicillium* spp., *Alternaria* spp., and *Cladosporium* spp. are included thus imposing a serious health threat on librarians, conservators, and scholars who might be in contact with these documents (Cappitelli & Sorlini, 2005; Kadaifciler, 2017; Mesquita et al., 2009). Therefore, it is important for all users to be aware of this issue and adequate precautions should be taken when handling documents infested with these molds.

Analysis of the bacterial diversity retrieved by culture‐dependent methods, revealed 15 phylotypes, belonging to Firmicutes and Actinobacteria. These phylotypes were closely related to: *Staphylococcus* sp. (2), *Kocuria* sp.(1) *Paenibacillus provencensis*(1)*, S. hominis*(2)*, S. epidermidis* (4)*, S. pasteuri* (1)*, Micrococcus luteus* (2)*, M. yunnanensis* (1), and *Bacillus foraminis* (1) (Table 2).

**Table 2:** Bacteria isolated and identified with culture‐dependent methods

Strain/Genbank accession number	Paper document	Closest homologue accession number & sequence similarity (% identity)
BS1/KC492510	Sample 1	NR_044179.1 *Paenibacillus provencensis* (100%)
BS10/KC492511	Sample 3	JN615458.1 *Kocuria* sp. (100%)
BS11/KC492512	Sample 2	HE578786.1 *Staphylococcus hominis* (100%)
BS12/KC492513	Sample 1	FR799429.1 *Micrococcus luteus* (100%)
BS2/KC492514	Sample 1	FR775755.1 *Staphylococcus epidermidis* (99%)
BS3/KC492515	Sample 1	JN944739.1 *Staphylococcus hominis* (100%)
BS4/KC492516	Sample 3	AB681292.1 *Staphylococcus epidermidis* (100%)
BS5/KC492517	Sample 5	FR750973.1 *Micrococcus luteus* (100%)
BS6/KC492518	Sample 1	HM163530.1 *Bacillus foraminis* (99%)
BS7/KC492519	Sample 4	HQ663910.1 *Micrococcus yunnanensis* (100%)
BS9/KC492521	Sample 3	AB681292.1 *Staphylococcus epidermidis* (100%)
CS10/KC492522	Sample 4	HF564648.1 *Staphylococcus epidermidis* (100%)
CS11/KC492523	Sample 2	JX994109.1 *Staphylococcus pasteuri* (100%)
CS8/KC492524	Sample 5	HQ436427.1 *Staphylococcus* sp. (100%)
CS9/KC492525	Sample 1	JQ522974.1 *Staphylococcus* sp. (100%)

Most bacterial phylotypes (9 out of 15) isolated in this study belong to *Staphylococcus* spp. and their presence on document surface could be attributed to human contact since they are part of human skin microbiome (Baviera et al., 2014). Actinobacteria such as *M. luteus*,* M. yunnanensis,* and *Kocuria sp*. were among other isolated phylotypes. Interestingly, *Micrococcus* spp. and *Kocuria* spp. were the dominant isolated bacteria (58%) from indoor air samples of storerooms at the Auschwitz‐Birkenau museum (Niesler et al., 2010). *M. yunnanensis* was isolated from a 13th‐century historical document in Poland, whereas *M. luteus* is known to be implicated in biodeterioration of cultural objects due to proteolytic properties (Lech, 2016). Furthermore, *Kocuria* spp. have been isolated from archival items in previous studies (Lech, 2016; Puškárová et al., 2016) but their implication in biodeterioration was not clear. Recently, it has been shown that the lignocellulose depolymerizing multi‐enzyme complex, comprising of lignin peroxidase, xylanases, and cellulases were present in *Kocuria* spp. isolated from *Eucheuma cottonii,* indicating for the first time a putative contribution of these bacteria in paper biodeterioration (Satheeja Santhi, Bhagat, Saranya, Govindarajan, & Jebakumar, 2014).

**Figure 3:**
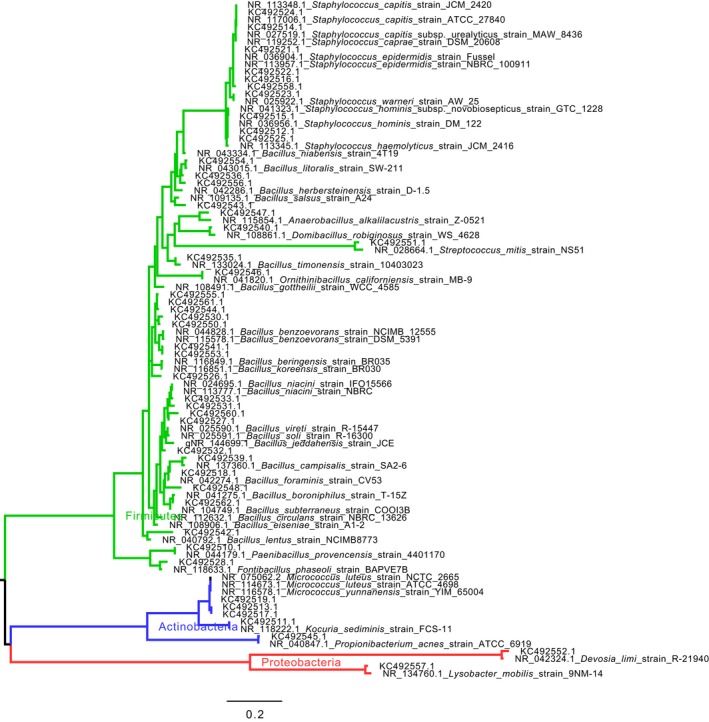
Maximum Likelihood Phylogenetic tree of Bacterial 16rRNA sequences fragments identified in this study, together with closely related sequences from publicly available sequence databases, retrieved by MOLE‐BLAST

**Figure 4:**
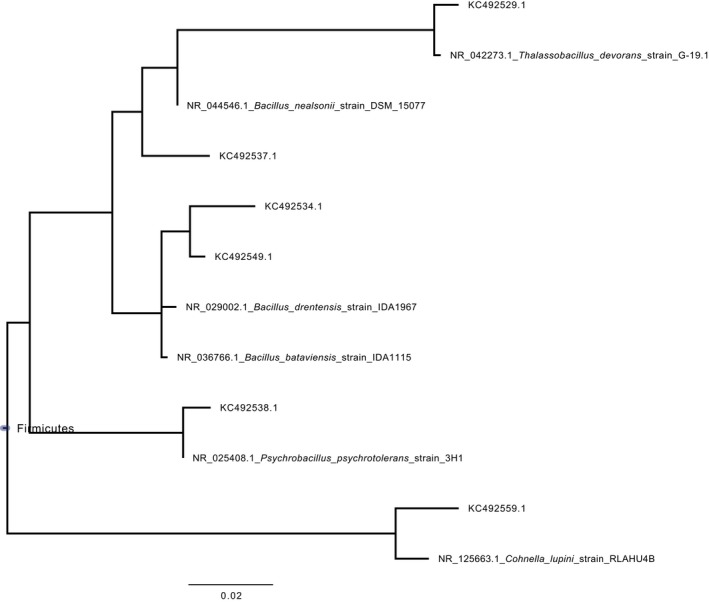
Maximum Likelihood Phylogenetic tree of Bacterial 16rRNA sequences fragments identified in this study, together with closely related sequences from publicly available sequence databases, retrieved by MOLE‐BLAST. This tree was based on an alignment of sequence fragments that had little or no overlap with sequence fragments used for the Phylogenetic tree of Figure 3

Sequence analysis of 16S rRNA gene clone library of the paper sample 4 revealed that most bacterial phylotypes belonged to *Bacillus* group (81.4%). Nevertheless, other Firmicutes such as *Paenibacillus* sp., *Halobacillus* sp., *Ornithinibacillus* sp., *Anaerobacillus* sp., *Streptococcus* sp., *Staphylococcus* sp., and *Cohnella* sp. were represented but less frequently (12.9%). Actinobacteria were represented by *Propionibacterium* sp., and Proteobacteria were represented by *Devosia* sp. (Alpha‐proteobacteria) and *Lysobacter* sp. (Gamma‐proteobacteria) but even less frequently (1.9% and 3.8%, respectively). Although library clone coverage based on Good's C estimator was not as high as it was for ITS clone libraries, species richness evaluated by Chao1 and Shannon indexes revealed quite high bacterial diversity (Table 3). The phylogenetic trees presented in Figures 3 and 4 depict the phylogenetic proximity between the bacterial phylotypes retrieved in this study and their most closely related bacteria in the MOLE‐BLAST database. From the combination of the two bacterial trees, 45 sequences were within the Firmicutes group, with 10 of them within the *Staphylococcus* genus and 35 of them within the *Bacillus* group. In Actinobacteria, three sequences were within the *Micrococcus* genus, one sequence was very closely related to *Kocuria sediminis* and one very closely related to *Propionibacterium acnes*. In Proteobacteria, two sequences were very closely related to *Devosia limi* and *Lysobacter mobilis,* respectively.

**Table 3:** Library coverage and species richness estimation in ITS and 16S rRNA gene clone libraries

Parameters	Fungi (Sample 1)	Fungi (Sample 4)	Bacteria (Sample 4)^b^
Total no. of OTUs^a^	2	3	17
% of library coverage	97.7	94.6	74.5
Chao1 species estimator	2	3	56
Shannon index	1.11	1.28	2.08

a OTUs at 3% of sequence difference.

b Values for bacteria were estimated according to Schloss et al. (2009).

Most bacterial phylotypes retrieved with culture‐independent methods belonged to the *Bacillus* group which are often associated with deterioration of archival items such as historical documents and photographs (Kraková et al., 2012; Lech, 2016; Piñar et al., 2015; Puškárová et al., 2016). Next to *Bacillus*, bacteria belonging to closely related genera such as *Paenibacillus*,* Halobacillus Ornithinibacillus,* and *Anaerobacillus* were identified but less frequently. Interestingly, *Halobacillus* spp. were identified in deteriorated wall paintings of the Cathrine chapel at Herberstein castle (Piñar et al., 2001) and the St. Virgil chapel in Vienna, both dating back to the 14th century (Ripka, Denner, Michaelsen, Lubitz, & Piñar, 2006). Identified bacterial phylotypes closely related to *Staphylococcus* sp., *Streptococcus* sp., and *Propionibacterium* sp. could be attributed to human skin contact as previously described (Principi et al., 2011). Moreover, identified proteobacterial phylotypes were closely related to *Devosia* sp. and *Lysobacter* sp. Recently, *L. dokdonensis* has been isolated from a biodeteriorated paper document dating back to the 18th century and it has shown cellulolytic and proteolytic activity (Kraková et al., 2012). *Devosia* spp. often display endophytic behavior and they colonize tree roots and trunks. Therefore, they might consist part of the microbiome present in trees used for papermaking. Their presence in biodeteriorated archaeological wood samples has been demonstrated previously (Landy, Mitchell, Hotchkiss, & Eaton, 2008; Nikolouli, Pournou, McConnachie, Tsiamis, & Mossialos, 2016).

Recently, next generation sequencing (NGS) has been employed to assess microbial diversity in cultural heritage as a cutting‐edge culture‐independent method (Adamiak, Otlewska, Tafer, Lopandic, Gutarowskaa, Sterflingerb, & Piñar, 2017; Kraková et al., 2016; Ogawa, Celikkol‐Aydin, Gaylarde, Baptista Neto, & Beech, 2017). Although NGS has very high sensitivity and reveals the vast diversity of the sampled microbiota this does not necessary reflects a putative involvement of very low abundance phylotypes in the biodeterioration process. A recent study of the prokaryotic diversity in biodeteriorated archaeological wood has employed two different culture‐independent methods: 16S rRNA gene clone libraries and NGS (Nikolouli et al., 2016). Comparison of these two approaches has revealed that only three bacterial phylotypes were solely identified by NGS (Nikolouli et al., 2016). Therefore, in this study ITS and 16S rRNA gene clone libraries have been employed to retrieve the dominant microbial phylotypes present in biodeteriorated historical manuscripts.

Certain fungi and bacteria retrieved in this study are well‐known biodeteriogenic agents in accordance with previous studies. New or less‐known fungi and bacteria implicated in paper degradation have been described, indicating that particular ambient conditions, substrate chemistry, or even location might influence the composition of colonizing microbiota. The findings of this study might be useful as a guideline in designing and developing target‐specific monitoring schemes thus minimizing biodeterioration of valuable historical documents and the health impact on all users.

## CONFLICT OF INTEREST

Authors declare that they have no conflict of interest.

## References

[mbo3596-bib-0001] Adamiak, J. , Otlewska, A. , Tafer, H. , Lopandic, K. , Gutarowskaa, B. , Sterflingerb, K. , & Piñar, G. (2017). First evaluation of the microbiome of built cultural heritage by using the Ion Torrent next generation sequencing platform. International Biodeterioration & Biodegradation, 10.1016/j.ibiod.2017.01.040

[mbo3596-bib-0002] Arai, H. (2000). Foxing caused by fungi: Twenty‐five years of study. International Biodeterioration and Biodegradation, 46, 181–188. 10.1016/S0964-8305(00)00063-9

[mbo3596-bib-0003] Baviera, G. , Leoni, M. C. , Capra, L. , Cipriani, F. , Longo, G. , Maiello, N. , … Galli, E. (2014). Microbiota in healthy skin and in atopic eczema. BioMed Research International, 2014, 436921. 25126558 10.1155/2014/436921 PMC4122000

[mbo3596-bib-0004] Borrego, S. , Lavin, P. , Perdomo, I. , Gómez de Saravia, S. , & Guiamet, P. (2012). Determination of indoor air quality in archives and biodeterioration of the documentary heritage. ISRN Microbiology, 2012, 680598. 23762758 10.5402/2012/680598 PMC3671707

[mbo3596-bib-0005] Borrego, S. , Molina, A. , & Santana, A. (2015). Mold on stored photographs and maps: A case study. Topics in Photographic Preservation, 16, 109–120.

[mbo3596-bib-0006] Borrego, S. , & Perdomo, I. (2014). Characterization of air mycobiota in two repositories of the National Archives of the Republic of Cuba. Revista Iberoamericana de Micología, 31(3), 182–187. 10.1016/j.riam.2013.09.004 24071642

[mbo3596-bib-0007] Cappitelli, F. , Pasquariello, G. , Tarsitani, G. , & Sorlini, C. (2010). Scripta manent? Assessing microbial risk to paper heritage. Trends in Microbiology, 18, 538–542. 10.1016/j.tim.2010.09.004 20971645

[mbo3596-bib-0008] Cappitelli, F. , & Sorlini, C. (2005). From papyrus to compact disc: The microbial deterioration of documentary heritage. Critical Reviews in Microbiology, 31, 1–10. 10.1080/10408410490884766 15839400

[mbo3596-bib-0009] Chao, A. (1984). Nonparametric estimation of the number of classes in a population. Scandinavian Journal of Statistics, 11, 265–270.

[mbo3596-bib-0010] Chao, A. (1987). Estimating the population size for capture‐recapture data with unequal catchability. Biometrics, 43, 783–791. 10.2307/2531532 3427163

[mbo3596-bib-0011] Choi, S. (2007). Foxing on paper: A literature review. Journal of the American Institute for Conservation, 46, 137–152. 10.1179/019713607806112378

[mbo3596-bib-0012] Crous, P. W. , Braun, U. , Wingfield, M. J. , Wood, A. R. , Shin, H. D. , Summerell, B. A. , … Groenewald, J. Z. (2009). Phylogeny and taxonomy of obscure genera of microfungi. Persoonia, 22, 139–161. 10.3767/003158509X461701 20198145 PMC2789545

[mbo3596-bib-0013] Dai, Y. C. , Xu, M. Q. , Yang, Z. , & Jiang, M. L. (2008). Wood‐decaying fungi on timber or wooden constructions in China. Forest Research, 21, 44–49.

[mbo3596-bib-0014] Darriba, D. , Taboada, G. L. , Doallo, R. , & Posada, D. (2012). jModelTest 2: More models, new heuristics and parallel computing. Nature Methods, 9, 772 10.1038/nmeth.2109 PMC4594756 22847109

[mbo3596-bib-0015] De Paolis, M. R. , & Lippi, D. (2008). Use of metabolic and molecular methods for the identification of a Bacillus strain isolated from paper affected by foxing. Microbiological Research, 163, 121–131. 10.1016/j.micres.2007.06.002 17686620

[mbo3596-bib-0016] Edgar, R. C. (2004). MUSCLE: Multiple sequence alignment with high accuracy and high throughput. Nucleic Acids Research, 32, 1792–1797. 10.1093/nar/gkh340 15034147 PMC390337

[mbo3596-bib-0017] Edgar, R. C. , Haas, B. J. , Clemente, J. C. , Quince, C. , & Knight, R. (2011). UCHIME improves sensitivity and speed of chimera detection. Bioinformatics, 27, 2194–2200. 10.1093/bioinformatics/btr381 21700674 PMC3150044

[mbo3596-bib-0018] El Bergadi, F. , Laachari, F. , Elabed, S. , Mohammed, I. H. , & Ibnsouda, S. K. (2014). Cellulolytic potential and filter paper activity of fungi isolated from ancients manuscripts from the Medina of Fez. Annals of Microbiology, 64, 815–822. 10.1007/s13213-013-0718-6

[mbo3596-bib-0020] Good, I. J. (1953). The population frequencies of species and the estimation of population parameters. Biometrika, 43, 45–63.

[mbo3596-bib-0021] Gouy, M. , Guindon, S. , & Gascuel, O. (2010). SeaView Version 4: A multiplatform graphical user interface for sequence alignment and phylogenetic tree building. Molecular Biology and Evolution, 27, 221–224. 10.1093/molbev/msp259 19854763

[mbo3596-bib-0022] Guiamet, P. S. , Borrego, S. , Lavin, P. , Perdomo, I. , & Gómez de Saravia, S. (2011). Biofouling and biodeterioration in material stored at Historical Archive of the Museum of La Plata, Argentine and at the National Archives of the Republic of Cuba. Colloid Surface B, 85(2), 229–234. 10.1016/j.colsurfb.2011.02.031 21439796

[mbo3596-bib-0024] Jacob, S. M. , Bhagwat, A. M. , & Kelkar‐Mane, V. (2015). Bacillus species as an intrinsic controller of fungal deterioration of archival documents. International Biodeterioration and Biodegradation, 104, 46–52. 10.1016/j.ibiod.2015.05.001

[mbo3596-bib-0025] Kadaifciler, D. G. (2017). Bioaerosol assessment in the library of Istanbul University and fungal flora associated with paper deterioration. Aerobiologia, 33, 151–166. 10.1007/s10453-016-9457-z

[mbo3596-bib-0026] Kraková, L. , Chovanová, K. , Selim, S. A. , Simonovicová, A. , Puskarová, A. , Maková, A. , & Pangallo, D. (2012). A multiphasic approach for investigation of the microbial diversity and its biodegradative abilities in historical paper and parchment documents. International Biodeterioration and Biodegradation, 70, 117–125. 10.1016/j.ibiod.2012.01.011

[mbo3596-bib-0027] Kraková, L. , Šoltys, K. , Otlewska, A. , Pietrzak, K. , Purkrtová, S. , Savická, D. , … Pangallo, D. (2016). Comparison of methods for identification of microbial communities in book collections: Culture‐dependent (sequencing and MALDI‐TOF MS) and culture‐independent (Illumina MiSeq). International Biodeterioration & Biodegradation, 10.1016/j.ibiod.2017.02.015

[mbo3596-bib-0028] Landy, E. T. , Mitchell, J. I. , Hotchkiss, S. , & Eaton, R. A. (2008). Bacterial diversity associated with archaeological waterlogged wood: Ribosomal RNA clone libraries and denaturing gradient gel electrophoresis (DGGE). International Biodeterioration and Biodegradation, 61, 106–116. 10.1016/j.ibiod.2007.07.007

[mbo3596-bib-0030] Lavin, P. , de Saravia, S. G. , & Guiamet, P. S. (2014). An environmental assessment of biodeterioration in document repositories. Biofouling, 30, 561–569. 10.1080/08927014.2014.897334 24708295

[mbo3596-bib-0031] Lavin, P. , de Saravia, S. G. , & Guiamet, P. (2016). *Scopulariopsis* sp. and *Fusarium* sp. in the documentary heritage: evaluation of their biodeterioration ability and antifungal effect of two essential oils. Microbial Ecology, 71, 628–633. 10.1007/s00248-015-0688-2 26500067

[mbo3596-bib-0032] Lech, T. (2016). Evaluation of a parchment document, the 13th century incorporation charter for the city of krakow, poland, for microbial hazards. Applied and Environment Microbiology, 82, 2620–2631. 10.1128/AEM.03851-15 PMC4836425 26896133

[mbo3596-bib-0034] Mesquita, N. , Portugal, A. , Videira, S. , Rodrıguez‐Echeverri, A. A. , Bandeira, A. M. L. , Santos, M. J. A. , & Freitas, H. (2009). Fungal diversity in ancient documents: A case study on the Archive of the University of Coimbra. International Biodeterioration and Biodegradation, 63, 626–629. 10.1016/j.ibiod.2009.03.010

[mbo3596-bib-0035] Michaelsen, A. , Piñar, G. , & Pinzari, F. (2010). Molecular and microscopical investigation of the microflora inhabiting a deteriorated Italian manuscript dated from the thirteenth century. Microbial Ecology, 60, 69–80. 10.1007/s00248-010-9667-9 20449583 PMC2917558

[mbo3596-bib-0036] Michaelsen, A. , Pinzari, F. , Barbabietola, N. , & Piñar, G. (2013). Monitoring the effects of different conservation treatments on paper‐infecting fungi. International Biodeterioration and Biodegradation, 84, 333–341. 10.1016/j.ibiod.2012.08.005 24092956 PMC3728566

[mbo3596-bib-0037] Montemarini‐Corte, A. , Ferroni, A. , & Salvo, V. S. (2003). Isolation of fungal species from test samples and maps damaged by foxing, and correlation between these species and the environment. International Biodeterioration and Biodegradation, 51, 167–173. 10.1016/S0964-8305(02)00137-3

[mbo3596-bib-0038] Mueller, G. M. , & Schmit, J. P. (2007). Fungal biodiversity: What do we know? What can we predict? Biodiversity and Conservation, 16, 1–5. 10.1007/s10531-006-9117-7

[mbo3596-bib-0039] Niesler, A. , Górny, R. L. , Wlazło, A. , Łudzeń‐Izbińska, B. , Ławniczek‐Wałczyk, A. , Gołofit‐Szymczak, M. , … Anczyk, E. (2010). Microbial contamination of storerooms at the Auschwitz‐Birkenau Museum. Aerobiologia, 26, 125–133. 10.1007/s10453-009-9149-z

[mbo3596-bib-0040] Nikolouli, K. , & Mossialos, D. (2012). Bioactive compounds synthesized by non‐ribosomal peptide synthetases and type‐I polyketide synthases discovered through genome‐mining and metagenomics. Biotechnology Letters, 34, 1393–1403. 10.1007/s10529-012-0919-2 22481301

[mbo3596-bib-0041] Nikolouli, K. , Pournou, A. , McConnachie, G. , Tsiamis, G. , & Mossialos, D. (2016). Prokaryotic diversity in biodeteriorated wood coming from the Bükkábrány fossil forest. International Biodeterioration and Biodegradation, 108, 181–190. 10.1016/j.ibiod.2015.12.023

[mbo3596-bib-0042] Ogawa, A. , Celikkol‐Aydin, S. , Gaylarde, C. , Baptista Neto, J. A. , & Beech, I. (2017). Microbial communities on painted wet and dry external surfaces of a historic fortress in Niteroi, Brazil. International Biodeterioration and Biodegradation, 123, 164–173. 10.1016/j.ibiod.2017.06.018

[mbo3596-bib-0043] Olennikov, D. N. , Agafonova, S. V. , Penzina, T. A. , & Borovskiî, G. B. (2014). Fatty acid composition of fourteen wood‐decaying basidiomycete species growing in permafrost conditions. Records of Natural Products, 8, 184–188.

[mbo3596-bib-0044] Paiva de Carvalho, H. , Mesquita, N. , Trovão, J. , Peixoto da Silva, J. , Rosa, B. , Martins, R. , … Portugal, A. (2016). Diversity of fungal species in ancient parchments collections of the Archive of the University of Coimbra. International Biodeterioration and Biodegradation, 108, 57–66. 10.1016/j.ibiod.2015.12.001

[mbo3596-bib-0045] Pasquarella, C. , Saccani, E. , Sansebastiano, G. E. , Ugolotti, M. , Pasquariello, G. , & Albertini, R. (2012). Proposal for a biological environmental monitoring approach to be used in libraries and archives. Annals of Agricultural and Environmental Medicine, 19, 209–212. 22742789

[mbo3596-bib-0046] Piñar, G. , Ramos, C. , Rölleke, S. , Schabereiter‐Gurtner, C. , Vybiral, D. , Lubitz, W. , & Denner, E. B. (2001). Detection of indigenous Halobacillus populations in damaged ancient wall paintings and building materials: Molecular monitoring and cultivation. Applied and Environment Microbiology, 67, 4891–4895. 10.1128/AEM.67.10.4891-4895.2001 PMC93245 11571198

[mbo3596-bib-0047] Piñar, G. , Tafer, H. , Sterflinger, K. , & Pinzari, F. (2015). Amid the possible causes of a very famous foxing: Molecular and microscopic insight into Leonardo da Vinci's self‐portrait. Environmental Microbiology Reports, 7, 849–859. 26111623 10.1111/1758-2229.12313 PMC4959533

[mbo3596-bib-0048] Pinzari, F. , Cialei, V. , & Barbabietola, N. (2010). Measurement of the micro‐aeroflora deteriorating potentialities in the indoor environments. e‐Preserv Sci, 7, 29–34.

[mbo3596-bib-0049] Pinzari, F. , Pasquariello, G. , & De Mico, A. (2006). Biodeterioration of paper: A SEM study of fungal spoilage reproduced under controlled conditions. Macromolecular Symposium, 238, 57–66. 10.1002/(ISSN)1521-3900

[mbo3596-bib-0050] Polo, A. , Cappitelli, F. , Villa, F. , & Pinzari, F. (2017). Biological invasion in the indoor environment: The spread of *Eurotium halophilicu*m on library materials. International Biodeterioration and Biodegradation, 118, 34–44. 10.1016/j.ibiod.2016.12.010

[mbo3596-bib-0051] Principi, P. , Villa, F. , Sorlini, C. , & Cappitelli, F. (2011). Molecular studies of microbial community structure on stained pages of Leonardo da Vinci's Atlantic Codex. Microbial Ecology, 61, 214–222. 10.1007/s00248-010-9741-3 20811884

[mbo3596-bib-0052] Puškárová, A. , Bučková, M. , Habalová, B. , Kraková, L. , Maková, A. , & Pangallo, D. (2016). Microbial communities affecting albumen photography heritage: A methodological survey. Scientific Reports, 6, 20810. 26864429 10.1038/srep20810 PMC4749957

[mbo3596-bib-0053] Ripka, K. , Denner, E. B. M. , Michaelsen, A. , Lubitz, W. , & Piñar, G. (2006). Molecular characterisation of *Halobacillus* strains isolated from different medieval wall paintings and building materials in Austria. International Biodeterioration and Biodegradation, 58, 124–132. 10.1016/j.ibiod.2006.05.004

[mbo3596-bib-0054] Satheeja Santhi, V. , Bhagat, A. K. , Saranya, S. , Govindarajan, G. , & Jebakumar, S. R. D. (2014). Seaweed (*Eucheuma cottonii*) associated microorganisms, a versatile enzyme source for the lignocellulosic biomass processing. International Biodeterioration and Biodegradation, 96, 144–151. 10.1016/j.ibiod.2014.08.007

[mbo3596-bib-0055] Schloss, P. D. , Westcott, S. L. , Ryabin, T. , Hall, J. R. , Hartmann, M. , Hollister, E. B. , … Weber, C. F. (2009). Introducing mothur: Open‐source, platform‐independent, community‐supported software for describing and comparing microbial communities. Applied and Environment Microbiology, 75, 7537–7541. 10.1128/AEM.01541-09 PMC2786419 19801464

[mbo3596-bib-0056] Sequeira, S. , Cabrita, E. J. , & Macedo, M. F. (2012). Antifungals on paper conservation: An overview. International Biodeterioration and Biodegradation, 74, 67–86. 10.1016/j.ibiod.2012.07.011

[mbo3596-bib-0057] Sequeira, S. O. , Phillips, A. J. L. , Cabrita, E. J. , & Macedo, M. F. (2017). Antifungal treatment of paper with calcium propionate and parabens: Short‐term and long‐term effects. International Biodeterioration and Biodegradation, 120, 203–215. 10.1016/j.ibiod.2017.03.005

[mbo3596-bib-0058] Song, J. , Gu, J. , Zhai, Y. , Wu, W. , Wang, H. , Ruan, Z. , … Yan, Y. (2013). Biodegradation of nicosulfuron by a *Talaromyces flavus* LZM1. Bioresource Technology, 140, 243–248. 10.1016/j.biortech.2013.02.086 23707911

[mbo3596-bib-0059] Spilker, T. , Coenye, T. , Vandamme, P. , & LiPuma, J. J. (2004). PCR‐based assay for differentiation of *Pseudomonas aeruginosa* from other pseudomonas species recovered from cystic fibrosis patients. Journal of Clinical Microbiology, 42, 2074–2079. 10.1128/JCM.42.5.2074-2079.2004 15131172 PMC404678

[mbo3596-bib-0060] Štafura, A. , Nagy, Š. , Bučková, M. , Puškárová, A. , Lucia, Krakova , Čulík, M. , … Pangallo, D. (2017). The influence of microfilamentous fungi on wooden organ pipes: One year investigation. International Biodeterioration and Biodegradation, 121, 139–147. 10.1016/j.ibiod.2017.04.006

[mbo3596-bib-0061] Sterflinger, K. (2010). Fungi: Their role in deterioration of cultural heritage. Fungal Biology Reviews, 24, 47–55. 10.1016/j.fbr.2010.03.003

[mbo3596-bib-0062] Sterflinger, K. , & Pinzari, F. (2012). The revenge of time: Fungal deterioration of cultural heritage with particular reference to books, paper and parchment. Environmental Microbiology, 14, 559–566. 10.1111/j.1462-2920.2011.02584.x 22004478

[mbo3596-bib-0063] Tamura, K. , Dudley, J. , Nei, M. , & Kumar, S. (2007). MEGA4: Molecular Evolutionary Genetics Analysis (MEGA) software version 4.0. Molecular Biology and Evolution, 24, 1596–1599. 10.1093/molbev/msm092 17488738

[mbo3596-bib-0066] Yuan, Y. , Feng, H. , Wang, L. , Li, Z. , Shi, Y. , Zhao, L. , … Zhu, H. (2017). Potential of endophytic fungi isolated from cotton roots for biological control against verticillium wilt disease. PLoS ONE, 12, e0170557 10.1371/journal.pone.0170557 28107448 PMC5249208

[mbo3596-bib-0067] Zotti, M. , Ferroni, A. , & Calvinic, P. (2011). Mycological and FTIR analysis of biotic foxing on paper substrates. International Biodeterioration and Biodegradation, 65, 569–578. 10.1016/j.ibiod.2010.01.011

