# Supplementary material for: Microbial diversity in biodeteriorated Greek historical documents dating back to the 19th and 20th century: A case study
Source: Microbiologyopen. 2018 Feb 27;7(5):e00596. doi: 10.1002/mbo3.596 (PMC6182554; doi:10.1002/mbo3.596)
Supplement: Supplementary file 1 [file MBO3-7-e00596-s001.docx]

Table 1: Sequences of oligonucleotide primers used for the PCR amplification of the ITS regions in fungi and 16*S* rRNA gene of bacteria

| **PCR** | **Primer sequence (5’→3’)** | **Annealing temperature** | **Optimum PCR cycle number** |
| --- | --- | --- | --- |
| Fungi | ITS1: TCC GTA GGT GAA CCT GCG G^a^  ITS4: TCC TCC GCT TAT TGA TAT GC^a^  ITS1F: CTT GGT CAT TTA GAG GAA GTA A^b^  NLB4: GGA TTC TCA CCC TCT ATG AC^c^ | 55^o^C | 40 |
| Bacteria | 27F: AGAGTTTGATCMTGGCTCAG^d^  1492R:GGTTACCTTGTTACGACTT^e^ | 57^o^C | 30 |

^a^White et al. 1990

^b^Gardes and Bruns 1993

^c^[Martin and](http://www.ncbi.nlm.nih.gov/pubmed?term=Martin%20KJ%5BAuthor%5D&cauthor=true&cauthor_uid=15904497) [Rygiewicz 2005](http://www.ncbi.nlm.nih.gov/pubmed?term=Rygiewicz%20PT%5BAuthor%5D&cauthor=true&cauthor_uid=15904497)

^d^ Lane et al. 1991

^e^ Turner et al. 1999
